# Photodynamic inactivation of *Mycobacterium tuberculosis* by broad-spectrum visible light

**DOI:** 10.1007/s00253-026-13709-0

**Published:** 2026-01-23

**Authors:** Cartesio D’Agostini, Carla Prezioso, Paola Checconi, Valeria Camicia, Marco Pelliccioni, Loide Di Traglia, Marilena Minieri, Jacopo M. Legramante, Enrico Garaci, Dolores Limongi

**Affiliations:** 1 Laboratory of Clinical Microbiology, Policlinico Tor Vergata, Rome, Italy; 2 Department of Experimental Medicine, University of Rome Tor Vergata, Rome, Italy; 3 Department for the Promotion of Human Sciences and Quality of Life, San Raffaele University, Rome, Italy; 4 Laboratory of Microbiology, IRCCS San Raffaele Roma, Rome, Italy; 5 Unit of Laboratory Medicine, University Hospital Tor Vergata, Rome, Italy; 6 Department of Emergency, University Hospital Tor Vergata, Rome, Italy; 7 Department of Systems Medicine, University of Rome Tor Vergata, Rome, Italy; 8 San Raffaele Sulmona, Sulmona, Aquila, Italy

**Keywords:** *Mycobacterium tuberculosis* (Mtb), Photodynamic inactivation (PDI), Visible light irradiation (400–420 nm), Antimicrobial resistance (AMR), Photosensitizers (PS)

## Abstract

**Abstract:**

*Mycobacterium tuberculosis* (Mtb) is the causative agent of tuberculosis, a major infectious disease causing substantial global morbidity and mortality. The growing incidence of antibiotic-resistant strains highlights the urgency of identifying alternative strategies capable of targeting persistent and resistant forms of Mtb. This study evaluates the effectiveness of photodynamic inactivation (PDI) using visible light within the 400–420 nm range against a clinical Mtb isolate. The isolate was subjected to controlled LED exposure under biosafety level-3 conditions, with viability assessed via colony-forming unit counts. Results showed a significant reduction in Mtb viability, with > 99% (over 2-log) reduction observed after exposure to the tested visible light spectrum. These results provide a strong rationale for the clinical translation of visible light-based disinfection strategies, by indicating that visible light photoinactivation provides a practical, non-chemical alternative to conventional disinfectants and UV-based methods, reducing concerns about toxicity and operational limitations. This approach holds particular promise for healthcare environments, where it could contribute to reducing the environmental persistence of Mtb and, consequently, the risk of transmission. These results underscore the need for further investigation into light-based technologies as complementary tools to existing infection control measures, particularly in high-risk or inadequately sanitized settings.

**Key points:**

• *Visible light (Soret Band 400–420 nm) significantly reduces Mycobacterium tuberculosis (Mtb) viability*

• *No photosensitizers are used in the photodynamic inactivation (PDI) process*

• *LED-based visible light may help limit Mtb spread in clinical environments *

## Introduction

Tuberculosis (TB) remains one of the leading infectious causes of death worldwide. According to the World Health Organization (WHO 2023a, b), the incidence of TB has increased in recent years, from 10 million new cases in 2019 to approximately 10.8 million in 2023, underscoring how far current trends are from achieving the WHO “End TB” targets.

Forecasting models estimate that, even assuming a 2% annual decline in mortality, more than 30 million TB-related deaths may occur between 2020 and 2050, resulting in an estimated global economic loss of US$17.5 trillion (Silva et al. 2021). Although TB as a whole remains a major global killer, the spread of drug-resistant forms poses an additional and escalating threat: the Centers for Disease Control and Prevention (CDC 2023) project that up to 2.6 million deaths per year could occur by 2050 if the dissemination of resistant strains is not effectively contained.

Modern urban lifestyles contribute to increased time spent indoors, where microbial contamination is common (Leung et al. 2019; Dai et al. 2017).

Indoor environments—characterized by limited ventilation, high occupancy, and prolonged close contact—facilitate TB transmission by enabling the persistence of airborne droplet nuclei and increasing the likelihood of inhalation by susceptible individuals. In healthcare settings, persistent contamination by drug-resistant pathogens—including members of the WHO-defined ESKAPE group (*Enterococcus faecium*, *Staphylococcus aureus*, *Klebsiella pneumoniae*, *Acinetobacter baumannii*, *Pseudomonas aeruginosa*, and *Enterobacter* spp.)—is a major driver of healthcare-associated infections (HAIs), which affect up to 15% of hospitalized patients globally (Allegranzi et al. 2011; Hota 2004; Otter et al. 2013).

HAIs continue to represent a daily challenge in hospitals and clinics (WHO 2024), and although many are caused by drug-resistant bacteria, significant knowledge gaps persist regarding the true burden of hospital-associated resistant infections (HARIs) (Balasubramanian et al. 2023).

Among the major pathogens of concern, *Mycobacterium tuberculosis* (Mtb) exhibits distinct biological and genetic features. Unlike other ESKAPE pathogens, Mtb develops drug resistance primarily through spontaneous point mutations rather than horizontal gene transfer (HGT).

While its limited genetic diversity and low recombination rates constrain genetic exchange, these same features favor the clonal fixation and persistence of resistance mutations once they emerge, thereby sustaining the spread of resistant lineages (Sachan et al. 2023).

Another critical aspect of Mtb pathobiology is its ability to establish latent tuberculosis infection (LTBI), which is estimated to affect up to one quarter of the global population (WHO 2023b). Latent TB, an asymptomatic and dormant infection state, enables Mtb to evade immune detection and antibiotic treatment, further complicating control efforts.

In addition, the pathogen’s capacity to survive for prolonged periods on environmental surfaces is facilitated by its lipid-rich cell wall, which provides notable resistance to desiccation and to a broad range of chemical agents (Jarlier et al. 1994; Alderwick et al. 2007).

Of increasing concern is the global emergence and dissemination of multidrug-resistant (MDR) and extensively drug-resistant (XDR) TB strains—resistant to first-line drugs, any fluoroquinolone, and at least one injectable second-line agent (e.g., kanamycin).

The worldwide prevalence of MDR and XDR TB varies markedly by region, ranging from approximately 3–4% among new cases to over 20% among previously treated patients (Bu et al. 2023). Chemical disinfectants remain the mainstay for controlling environmental dissemination of Mtb; however, their efficacy is temporary, and repeated use may contribute to environmental toxicity and the selection of resistant organisms (Vandini et al. 2014; Nabi et al. 2020; Zhang et al. 2020; D’Accolti et al. 2021). Ultraviolet irradiation, although effective, is unsuitable for continuous use in clinical settings due to its photobiological hazards to human health (Demeersseman et al. 2023; Mmbando et al. 2024).

In this context, photodynamic inactivation (PDI) has emerged as a promising alternative. Most previous studies on the photoinactivation of mycobacteria, including Mtb, have relied on monochromatic light in combination with exogenous photosensitizers or precursors of porphyrin synthesis, such as 5-aminolevulinic acid, to enhance intracellular chromophore levels. PDI has demonstrated efficacy by generating reactive oxygen species (ROS) upon exposure to specific visible light wavelengths and even in the absence of exogenous photosensitizers has shown the ability to inactivate both drug-resistant and dormant mycobacteria (Shleeva et al. 2021).

Nevertheless, there remains a paucity of data regarding the efficacy of broad-spectrum, photosensitizer-free visible light approaches, particularly under clinically relevant conditions. The therapeutic use of light dates back to the nineteenth century, beginning with the work of Jacques-Louis Soret, who investigated the absorption spectra of biological materials and the effects of violet-blue light (400–420 nm) in the 1870s. Subsequently, Niels Ryberg Finsen, awarded the Nobel Prize in 1903, pioneered the medical application of ultraviolet (UV) light for treating lupus vulgaris, a cutaneous manifestation of TB. In the early twenty-first century, renewed interest arose in the use of visible blue-violet light, particularly at a wavelength of 405 nm, for its potent bactericidal properties. Unlike UV light, this wavelength demonstrates strong antimicrobial activity without the harmful side effects associated with UV exposure (Maclean et al. 2014). More recent studies have confirmed that pathogens exhibit wavelength-specific susceptibility, highlighting the targeted antimicrobial potential of 405 nm light (Amodeo et al. 2022; Harris 2023).

Based on this background, the present study aims to evaluate the in vitro efficacy of photodynamic inactivation (PDI) using a broader range of visible light wavelengths (400–420 nm) against a clinical strain of Mtb, to explore its potential as a safe, non-chemical disinfection strategy applicable to healthcare environments.

## Material and methods

### Strain source and ethical approval

This study was conducted using a previously frozen clinical isolate of Mtb, obtained from a patient during routine diagnostic procedures. The isolate is stored in an institutional strain collection at the Laboratory of Clinical Microbiology, Policlinico Tor Vergata, under an internal identification code (ID 14419507) and is available upon reasonable request, subject to ethical and biosafety regulations. The use of these anonymized biological materials for research purposes was approved by the Local Ethics Committee (Protocol No. 259.22). Informed consent was not required, as the study involved no identifiable human data and was authorized in accordance with institutional and national ethical regulations and the Declaration of Helsinki.

### Bacterial strain identification and susceptibility profile

A clinical isolate of Mtb (ID 14419507) obtained during routine diagnostic procedures was used for all experiments. Species identification was performed using MALDI-TOF mass spectrometry (Bruker Biotyper, Bruker Daltonics, Germany), with a reference library covering more than 180 *Mycobacterium* species, allowing reliable differentiation of *M. tuberculosis complex* from *non-tuberculous mycobacteria*. Molecular confirmation was obtained by PCR amplification of the IS6110 insertion element, specific to the *M. tuberculosis complex*. Drug susceptibility testing was conducted using the BD BACTEC MGIT 960 system, confirming that the isolate was drug-sensitive to first-line agents (isoniazid, rifampicin, ethambutol, and pyrazinamide).

### Thawing, decontamination, and strain preparation

Prior to use, frozen Mtb isolate was thawed and subjected to an initial decontamination step using N-acetyl-L-cysteine (NALC) as a mucolytic agent and 2% sodium hydroxide (NaOH) as a decontaminant, following CLSI guidelines (CLSI 2018). To verify the efficacy of this treatment and exclude the presence of non-mycobacterial contaminants, aliquots were plated in parallel onto Tryptic Soy Agar with 5% sheep blood (TSS) and Mueller–Hinton Agar with 5% sheep blood (MHS) and incubated at 35 ± 2 °C for up to 72 h, with readings at 24 and 48 h. No growth was observed during this period, confirming the absence of fast-growing contaminants. This incubation period was considered sufficient to detect rapid- and moderate-growing bacterial or fungal contaminants, while slow-growing mycobacteria were subsequently monitored on Middlebrook 7H11 agar over an 8-week incubation period.

An additional decontamination and purification step was subsequently performed using the BD BBL™ MycoPrep kit, according to the manufacturer’s instructions. The freshly prepared MycoPrep solution was added to the sample, vortexed for 30 s, and allowed to stand for 15 min at room temperature. A phosphate buffer was then added to reach a final volume of 50 mL, followed by gentle inversion. The sample was centrifuged at 3000 × g for 20 min at 4 °C, and the supernatant was decanted. The sediment was resuspended and streaked again on TSA and MHS blood agar plates for 24–48 h to confirm effective decontamination. The resulting suspension was finally plated on Liofilchem Middlebrook 7H11 agar for selective growth and quantitative colony enumeration.

Following decontamination, the selected Mtb strain was reactivated by inoculation into Middlebrook 7H9 broth and incubated at 37 °C under 5–10% CO₂ until reaching visible turbidity, corresponding to the early logarithmic growth phase (typically after 5–7 days). This step ensured restoration of metabolic activity prior to experimental exposure. Serial dilutions were then prepared to achieve a target concentration of approximately 4 × 10^3^ CFU/mL, as confirmed by colony enumeration on Middlebrook 7H11 agar after incubation. Viability and purity were verified by colony recovery and species identification using MALDI-TOF mass spectrometry (Bruker Biotyper).

### Antibiotic susceptibility testing

Following species confirmation, antibiotic susceptibility testing was performed using the proportion method on Middlebrook 7H11 agar to determine the minimum inhibitory concentrations (MICs) for first-line anti-tubercular drugs.

### Culture medium for light exposure experiments

To ensure compatibility with light-based treatment, Middlebrook 7H11 Agar (Liofilchem) was selected as the culture medium due to its stability under light exposure and specificity for Mtb. A comparative assessment was conducted to rule out any impact of the medium on bacterial growth, evaluating CFU counts, colony morphology, and growth time. No significant differences were observed compared to standard culture media, confirming that any observed effects were attributable solely to the light treatment.

Middlebrook 7H11 agar is a chemically stable, non-photosensitizing medium that does not contain chromophoric or photoreactive components susceptible to visible light degradation. Its photostability was further confirmed by exposing uninoculated plates to the same illumination conditions and subsequently inoculating them with fast-growing reference bacteria (*Staphylococcus aureus*), which exhibited normal growth rate and colony morphology compared to non-exposed controls. Uninoculated plates containing only sterile water were included in each experiment as sterility controls to confirm the absence of environmental or procedural contamination. In parallel, inoculated but non-irradiated plates served as growth controls for baseline CFU determination. After exposure to the microbicidal light sources, all plates were incubated at 35 ± 2 °C for up to 8 weeks in an atmosphere enriched with 5–10% CO₂. For data reporting, all results are expressed as CFU or log₁₀(CFU); these values specifically represent CFU per milliliter (CFU/mL), calculated based on the plated volume and the initial bacterial suspension.

### Light source

**Fig. 1:**
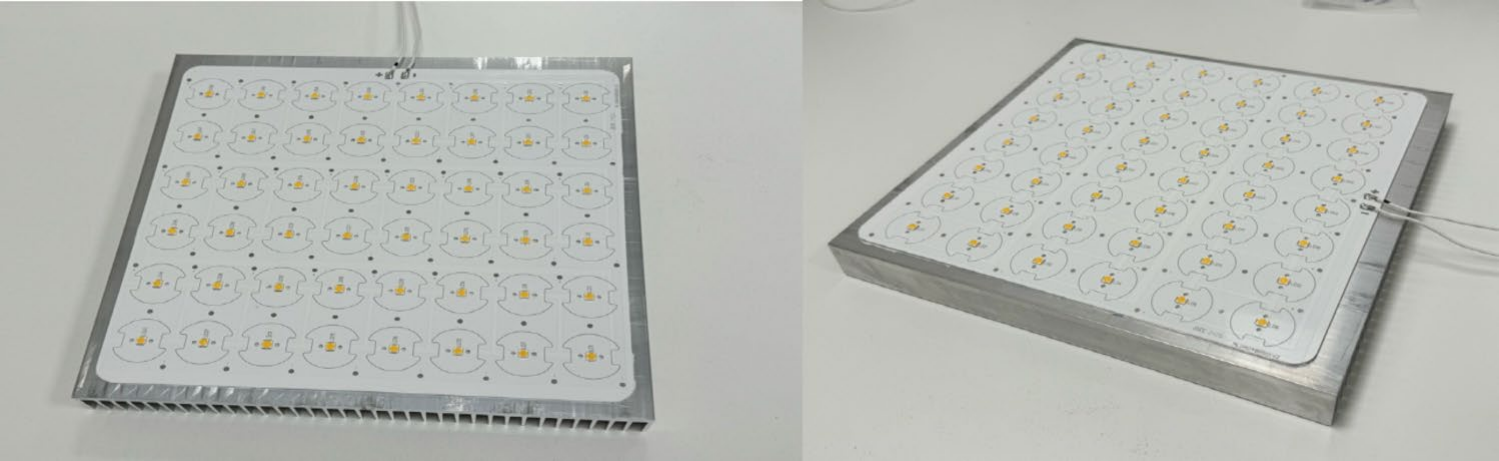
LED system used for photodynamic inactivation. This image shows the Biovitae® LED system, which emits visible light with a peak wavelength in the 400–420 nm range. The module served as the primary light source for microbial photoinactivation assays against clinical strain of Mtb. It was mounted at variable distances above the Petri dishes used for Mtb exposure and operated under standard laboratory conditions to ensure consistent exposure parameter

The antimicrobial light source was provided by Nextsense S.r.l., which owns the patented Biovitae® technology. The lighting system includes an array of 48 Opto PNDE-2LNE-8SR9 Biovitae® LEDs (CCT, 3966°K; CRI, 92; Duv, −0.0017) mounted on an aluminum PCB, driven by a 1200 mA constant current driver, with a power consumption of 53 W (Fig. 1).

**Fig. 2:**
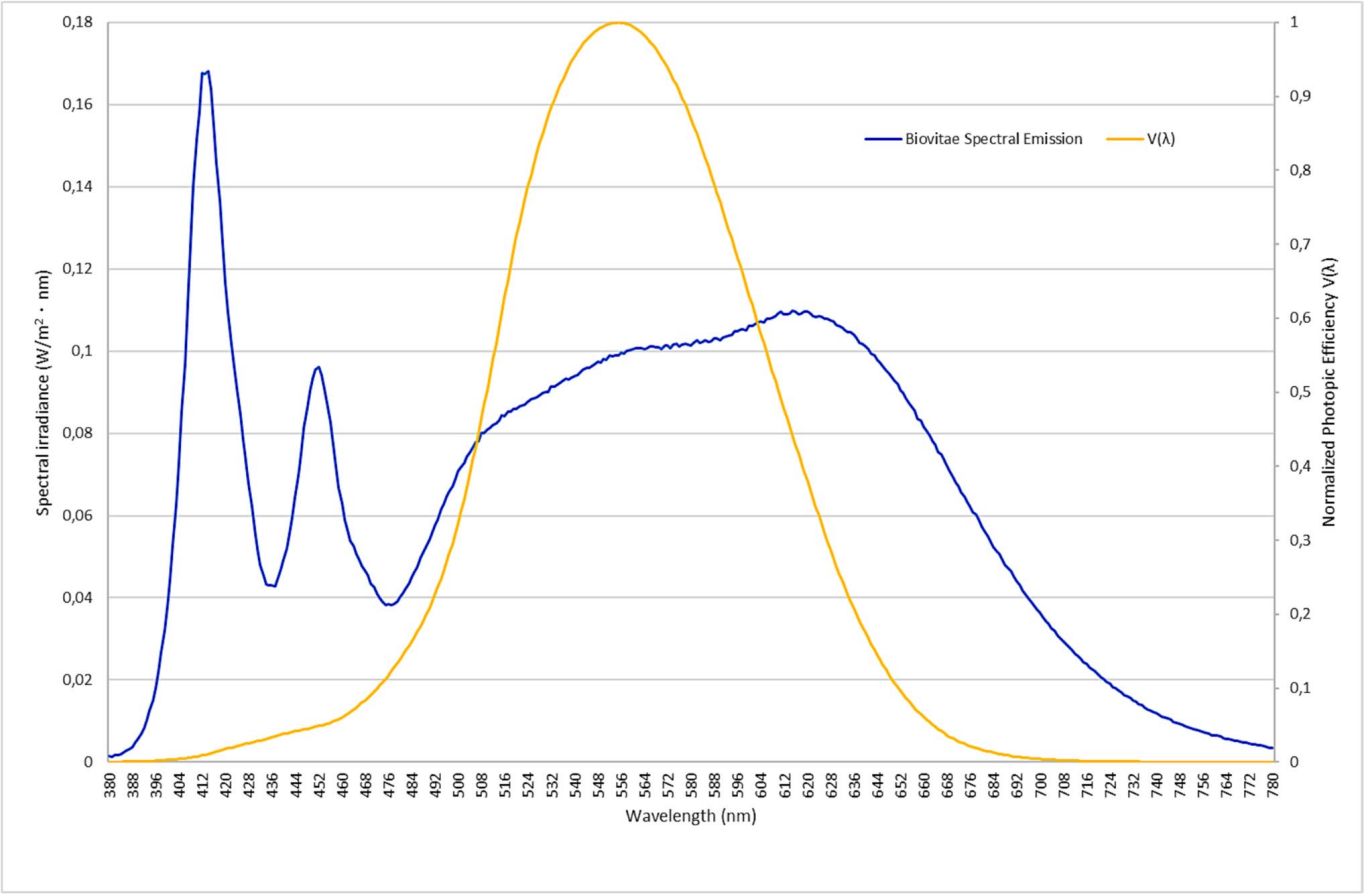
Spectral irradiance of the LED system and normalized human photopic sensitivity function *V*(*λ*). The graph displays the spectral emission of the LED system, characterized by a peak in the 400–420 nm range, alongside the normalized human photopic response function *V*(*λ*). The unweighted emission illustrates the energy distribution across the visible spectrum, while the photopic curve highlights the relative contribution of each wavelength to perceived brightness. The emission includes wavelengths within the Soret band, which are associated with the generation of ROS via endogenous chromophores, in the absence of external photosensitizers. Emission peaks include the 400–420 nm band. Irradiance was measured at plate level after 20-min warm-up

As illustrated in Fig. 2, the system emits a multi-peak spectrum within the visible range, including the Soret band, thereby overlapping with microbial chromophore absorption.

The light source, provided by the manufacturer Nextsense S.r.l. and previously validated according to IEC 62471 standards, was spectrally characterized using an Everfine PLA-30 spectroradiometer, after a 20-min stabilization period. Emission was confirmed in the 400–420 nm range, with irradiance levels of 1.166 mW/cm^2^ at 25 cm and 0.291 mW/cm^2^ at 50 cm. This blue-violet emission, characterized by multiple spectral peaks, differentiates the source from typical monochromatic light systems.

### Photobiological safety classification

The light source used in this study was previously assessed by the manufacturer for photobiological safety in accordance with IEC 62471:2006 and IEC 62471-7:2023 standards. Based on the provided documentation, the system is classified as Risk Group 1 (low risk), indicating minimal photobiological hazard during prolonged exposure under normal operating conditions. No additional safety tests were conducted in our laboratory.

### Light exposure treatment

All experiments were conducted under BSL-3 conditions inside a laminar flow hood maintained at 25 ± 2 °C. Middlebrook 7H11 Agar (Liofilchem) plates inoculated with Mtb were exposed to the light source at distances of 25 cm and 50 cm. At 25 cm, exposure durations of 60 and 120 min were tested; at 50 cm, exposures of 60, 120, and 180 min were performed. Each experimental condition was tested in three independent experiments, each performed with three technical replicates. Results were averaged accordingly.

To prevent background light interference, the internal lighting of the laminar flow hood was switched off during exposure. Ambient room lighting was kept at a minimal level, and the hood sash remained closed throughout each exposure to prevent any external light interference. Control plates (non-exposed but incubated under identical conditions) and sterile water controls were included in each experimental series.

### Irradiance measurement and dose calculation

The irradiance (E, W·cm⁻^2^) at the sample plane was measured directly at plate level using an Everfine PLA-30 spectroradiometer (350–800 nm range, wavelength accuracy ± 0.5 nm) after a 20-min warm-up of the LED source. Measurements were performed inside the biosafety cabinet under the same exposure conditions used for the experiments. The radiant exposure (H, J·cm⁻^2^) was calculated as *H* = *Ē* ×* t*, where *Ē* is the mean measured irradiance and* t* is the exposure duration in seconds. The measured irradiance values were 1.166 mW·cm⁻^2^ at 25 cm and 0.291 mW·cm⁻^2^ at 50 cm, corresponding to the energy doses reported in the “Results” section. The UV component was below the instrumental detection limit (no emission below 380 nm), confirming that only visible wavelengths contributed to the photoinactivation process.

### Plate number and arrangement during exposure

**Fig. 3:**
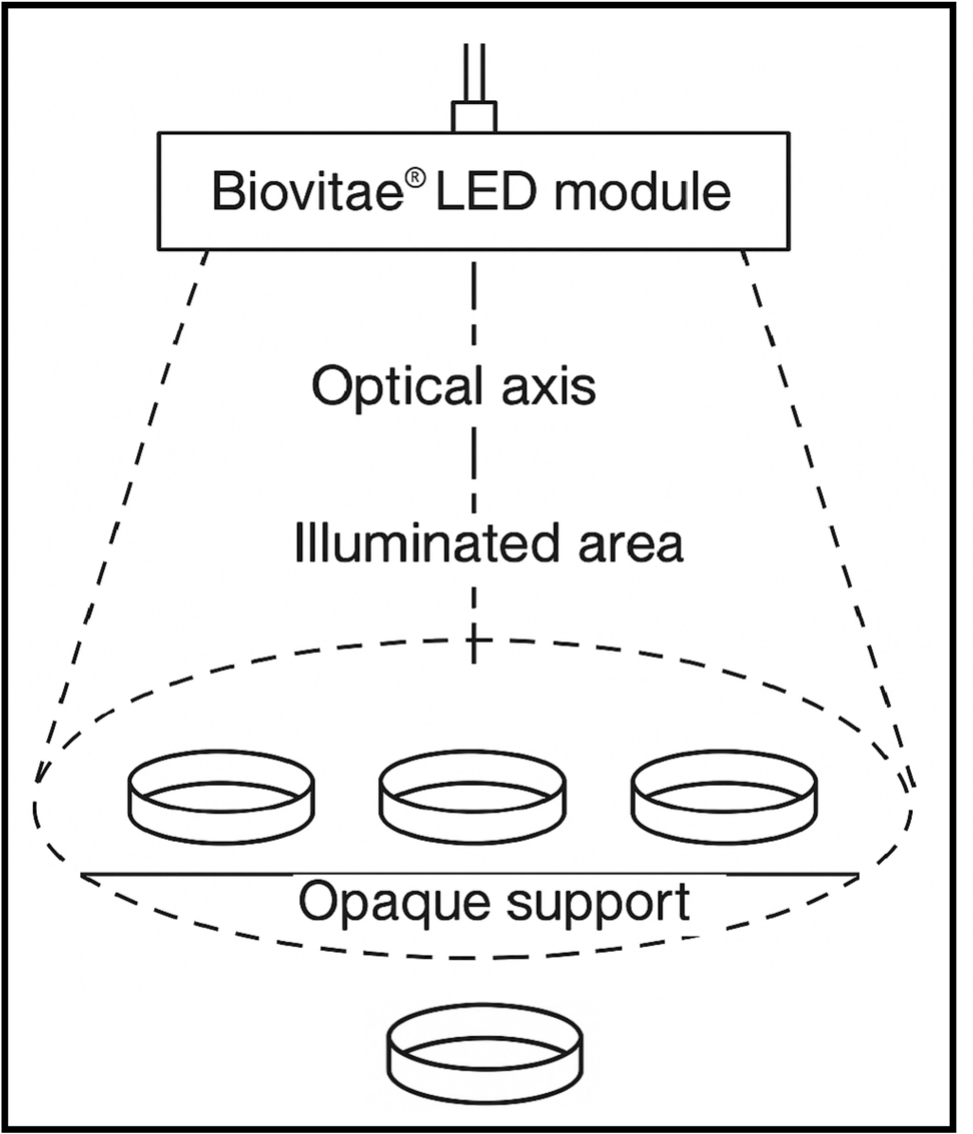
Schematic of plate arrangement under the Biovitae® LED source. Top-view schematic of the plate arrangement beneath the Biovitae® LED module (400–420 nm). Three Petri dishes were placed in a triangular configuration on a matte black support within the uniformly illuminated field (dashed circle) centered on the optical axis

For each exposure condition, three agar plates (Ø 90 mm; technical replicates) were irradiated simultaneously beneath the LED module. Plates were positioned on a matte, opaque, non-reflective black cardboard support to minimize reflections from the stainless-steel surfaces of the biosafety cabinet and to prevent diffuse light scattering. The plates were arranged in a configuration centered on the optical axis, with approximately 2–3 cm spacing between plates to avoid mutual shadowing. All exposures were performed with the internal illumination of the laminar flow hood switched off, ensuring that only the LED source contributed to the incident irradiance. No lateral shielding panels were employed, as irradiance mapping confirmed good spatial uniformity. Non-exposed control plates were kept outside the illuminated area within the same BSL-3 cabinet (Fig. 3).

Irradiance mapping was performed at plate level using an Everfine PLA-30 spectroradiometer, with measurements taken at five points (center and four quadrants) under each distance condition (25 cm and 50 cm). The recorded values showed minimal variation, confirming that the light field was spatially uniform across the exposure area.

### Statistical analysis

Colony-forming units (CFUs) were enumerated after incubation to assess the reduction in bacterial load. Reported results represent mean values from three independent experiments, each conducted in triplicate. Standard deviation (SD) and coefficient of variation (CV%) were calculated to assess intra-group variability. Data normality was assessed using the Shapiro–Wilk test. For normally distributed data, Student’s *t*-test was used; otherwise, the Mann–Whitney *U* test was applied. A *p*-value ≤ 0.05 was considered statistically significant. All statistical analyses were performed using GraphPad Prism software.

## Results

### Photoinactivation efficacy at 25 cm and at 50 cm

**Fig. 4:**
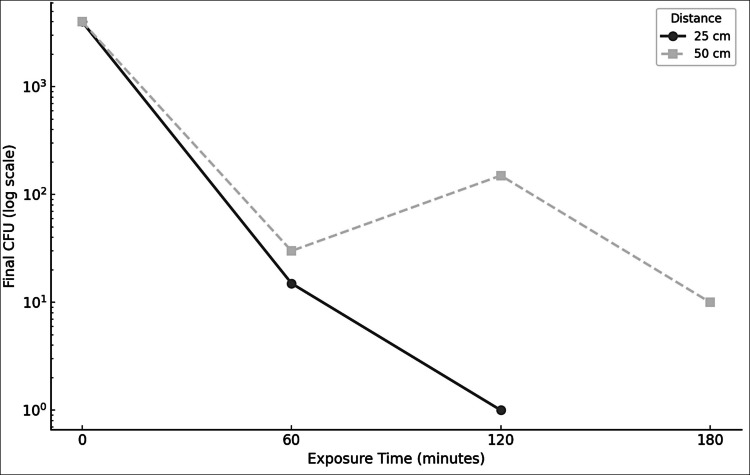
Reduction in Mtb viability as a function of exposure time and distance. The graph displays the mean colony-forming unit (CFU) counts of Mtb on the y-axis (log scale) in relation to exposure time to visible light (400–420 nm) on the x-axis (0, 60, 120, and 180 min). The two curves represent the reduction in bacterial load at two different distances from the light source: 25 cm (solid line) and 50 cm (dashed line). A faster and more pronounced CFU reduction is observed at 25 cm, while the 50 cm condition shows a more gradual decline, confirming the dose-dependent nature of the photoinactivation effect

Exposure of Mtb to visible light in the 400–420 nm range resulted in a marked, distance- and time-dependent reduction in bacterial viability. At 25 cm, the light source exhibited significant antimicrobial activity: after 60 min of exposure, the CFU count decreased from approximately 4000 to 15 (99.63% reduction; 2.43 log₁₀ reduction) and after 120 min to 1 CFU (99.98% reduction; 3.60 log₁₀ reduction). These reductions were consistently observed across three independent experiments, each performed in triplicate (Table 1, Fig. 4).

**Table 1:** Reduction of Mtb viability at 25 cm distance depending on exposure time to visible light (400–420 nm)

Exposure time (min)	Initial CFU	Final CFU (mean ± SD)	Reduction %	Log₁₀ reduction
0 (control)	4000	4000 ± 182.57	0.00%	0.00
60	4000	15 ± 1.15	99.63%	2.43
120	4000	1 ± 0.00	99.98%	3.60

Mean log₁₀(CFU) values and standard deviation for each exposure time at 25 cm. Data from three independent experiments

Despite the lower irradiance at 50 cm, exposures at this distance achieved reductions in CFU counts comparable to those observed at 25 cm. After 60 min, the CFU count decreased to 30 (99.25% reduction; 2.12 log₁₀ reduction from the initial value). After 120 min, CFUs were reduced to 150 (96.25% reduction; 1.43 log₁₀ reduction), and after 180 min, the CFU count further decreased to 10 (99.75% reduction; 2.60 log₁₀ reduction). Detailed results for individual replicates at each time point are provided (Table 2, Fig. 4).

**Table 2:** Reduction of Mtb viability at 50 cm distance depending on exposure time to visible light (400–420 nm)

Exposure time (min)	Initial CFU	Final CFU (mean ± SD)	Reduction %	Log₁₀ reduction
0 (control)	4000	4000 ± 182.57	0.00%	0.00
60	4000	30 ± 2.94	99.25%	2.12
120	4000	150 ± 14.33	96.25%	1.43
180	4000	10 ± 0.82	99.75%	2.60

Mean log₁₀(CFU) values and standard deviation for each exposure time at 50 cm. Data from three independent experiments

### Dose–response correlation

**Fig. 5:**
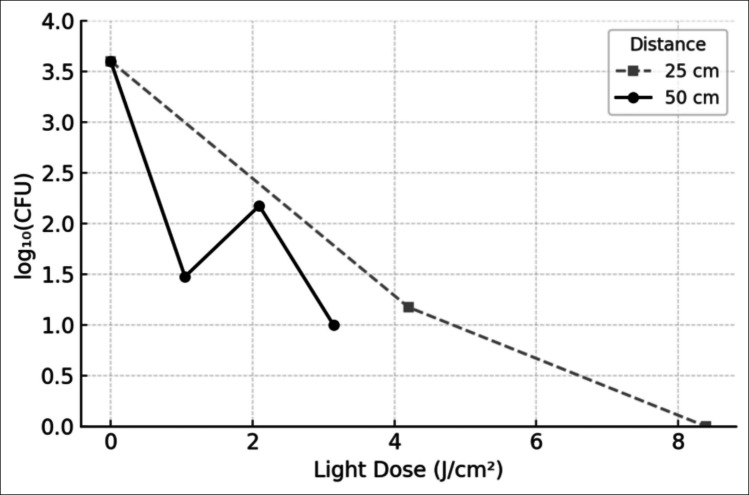
Residual viability of Mtb after exposure to visible light at different doses and distances. The graph illustrates the correlation between light dose (J/cm^2^) and the residual viability of Mtb, expressed as the base-10 logarithm of colony-forming units per milliliter [log₁₀(CFU/mL)]. The *x*-axis indicates the delivered energy dose, up to 8.4 J/cm^2^ at 25 cm and 3.1 J/cm^2^ at 50 cm. At 25 cm (gray dashed line with squares), the bacterial load decreased from an initial value of approximately 3.60 log₁₀(CFU/mL) (corresponding to 4000 CFU/mL) to approximately 2.18, 1.18, and finally 0.00 log₁₀(CFU/mL) with increasing exposure times, corresponding to log₁₀ reductions of 1.42, 2.42, and 3.60, respectively. At 50 cm (black solid line with circles), the residual CFU values were approximately 1.48, 2.18, and 1.00 log₁₀(CFU/mL), corresponding to log₁₀ reductions of 2.12, 1.42, and 2.60, respectively. The data highlight a non-linear dose–response relationship. Data represent mean values from three independent experiments, each performed in triplicate

To further explore the quantitative relationship between delivered light dose and bacterial survival, a dose–response analysis was performed. As illustrated in Fig. 5, a clear dose-dependent reduction in *Mtb* viability was observed. Notably, at 50 cm, an initial exposure of 60 min corresponding to a dose of 1.0476 J/cm^2^ resulted in a greater than > 2-log reduction (~2.12 log₁₀ reduction, 99.25% reduction), while the 120 min exposure at the same distance yielded a slightly lower (~1.43 log₁₀ reduction, 96.25% reduction). An unexpected result was recorded under the 50 cm at 120 min exposure condition, wherein the observed bacterial load reduction (96.25%) was lower than that measured after 60 min (99.25%) at the same distance.

### Validation of experimental consistency

Overall, these findings demonstrate that the bactericidal effect of visible light on Mtb is both distance- and dose-dependent, with higher radiant exposure producing greater CFU reduction. To confirm the reproducibility and internal consistency of the observed photoinactivation effects, variability was analyzed across three independent experiments performed in triplicate (Table 3). At 25 cm, an exposure of 60 min yielded a SD of 1.15 and a CV of 7.7%, compared to an SD of 182.57 and CV of 4.56% in unexposed controls (Table 3). At 120 min, the SD of exposed plates dropped to 0 (Table 1). At 50 cm, SDs after exposure were 2.94 (60 min), 14.33 (120 min), and 0.82 (180 min), all of which were lower than those observed in the unexposed control group (Table 3).

**Table 3:** Intra-experimental variability of CFU counts across experimental conditions

Condition	Mean CFU ± SD	CV%
Control (0 min)	4000 ± 182.57	4.56
25 cm–60 min	15 ± 1.15	7.70
25 cm–120 min	1 ± 0.00	0.00
50 cm–60 min	30 ± 2.94	9.80
50 cm–120 min	150 ± 14.33	9.55
50 cm–180 min	10 ± 0.82	8.20

Summary of experimental conditions: exposure times and distances tested for Mtb photoinactivation assays

## Discussion

The growing threat posed by multidrug-resistant Mtb (MDR/XDR-TB) demands new disinfection approaches that are both effective and safe. This study introduces a visible light-based antimicrobial solution using a broad LED emission in the 400–420 nm range, targeting intrinsic chromophores without the need for photosensitizing agents. Unlike conventional photodynamic therapy systems, mainly based on distinct monochromatic wavelengths of visible light, this platform leverages endogenous heme-related absorption (Soret band), thereby enhancing photobiological responses.

This effect is based on the ability of specific wavelengths to excite endogenous chromophores—such as porphyrins and flavins—naturally present within microbial cells. Upon excitation, these chromophores produce reactive oxygen species (ROS), including singlet oxygen and superoxide radicals, which disrupt vital cellular structures and lead to microbial inactivation. This mechanism, known as photodynamic inactivation (PDI), has been widely studied mainly using single-wavelength visible light sources in the range 400–500 nm, focusing on 405 nm (Mori et al. 2007; Maclean et al. 2009; Ghate et al. 2013; Kim et al. 2016; Maraccini et al. 2016).

Unlike the narrow bandwidth of 405 nm systems, the multi-peak spectrum within the visible light range, specifically targeting wavelengths between 400 and 420 nm, interacts with a wider array of endogenous chromophores found in different microbial species, enhancing the likelihood of ROS generation and microbial inactivation across a more diverse set of pathogens and environmental conditions. This spectral diversity translates to greater effectiveness since it not only triggers ROS generation more effectively than 405 nm sources, but also expands the antimicrobial action across a broader spectrum of endogenous chromophores. It improves efficacy against a wider variety of pathogens and under diverse environmental conditions, where monochromatic light might be less impactful or even ineffective.

In our study, the tested multi-peak spectral emission demonstrated a powerful antimicrobial effect against Mtb, a pathogen with a lipid-rich, highly impermeable cell wall that typically resists many physical and chemical treatments. This spectral emission penetrated these defenses and caused significant intracellular oxidative damage. For example, at 50 cm, a dose of 1.0476 J/cm^2^ was sufficient to achieve a > 2-log reduction in Mtb viability.

An unexpected finding was observed under the 50 cm/120 min exposure condition, where the CFU count was slightly higher than that recorded after 60 min at the same distance. The most plausible causes could include (i) intra-population phenotypic heterogeneity in Mtb populations, which may lead to transient recovery or photoadaptation effects among a minor fraction of surviving cells exposed to sub-lethal oxidative stress (Ghate et al. 2013; Kim et al. 2016; Shleeva et al. 2021), and (ii) minimal, intrinsic fluctuations in local irradiance distribution within the illumination field that can occur even under controlled and uniform exposure setups (Maclean et al. 2014; Amodeo et al. 2022). These small stochastic effects could contribute to slight variability in CFU enumeration at low viability levels but do not reflect systematic errors in plate arrangement or illumination uniformity. Importantly, this deviation does not affect the overall dose–response trend, which consistently demonstrates a robust and statistically significant reduction in Mtb viability with increasing exposure time and dose.

Although this discrepancy does not compromise the validity of the overall dose–response relationship, it highlights the importance of further investigations into the cellular and population-level dynamics occurring during exposure. Consequently, interpretation of the dose–log reduction relationship should be based on the general exposure pattern, rather than relying exclusively on individual time points.

Nevertheless, the level of efficacy is considerable when compared with previous studies using monochromatic 405 nm light sources, which required significantly higher doses—108 J/cm^2^ (Murdoch et al. 2012) and 120 J/cm^2^ (Guffey et al., 2013)—to inactivate surrogate *Mycobacterium* species such as *M. smegmatis* and *M. terrae*. These species are widely used as non-pathogenic models to approximate *Mtb* behavior due to similarities in cell wall structure and resistance traits (Shleeva et al. 2021). While the exact sensitivity thresholds may differ, this comparison highlights the enhanced efficiency of the multi-peak spectrum in achieving > 2-log reductions from the initial load at doses as low as 1.0476 J/cm^2^, over 100-fold lower than in earlier reports. Compared to conventional monochromatic approaches, the multi-peak LED system tested here demonstrated enhanced antimicrobial efficacy at substantially lower energy doses, without the use of exogenous photosensitizers. This advantage, together with a favorable photobiological safety profile, makes it particularly suitable for continuous application in real-world clinical environments. This higher efficacy not only reduces the exposure time needed for effective microbial inactivation but also minimizes energy consumption, providing a more sustainable and cost-effective solution.

Compared to UV systems, visible light wavelengths are non-cytotoxic to mammalian cells and are therefore considered safer for human exposure (Dai et al. 2012; Maclean et al. 2016, 2021). Although UV-C irradiation is a well-established and highly effective method for microbial inactivation, its use is limited to unoccupied environments because of its photobiological hazards and material degradation effects. Consequently, this study was not designed to compete with UV-based systems, but rather to evaluate visible light photoinactivation as a safer, continuously applicable, and photosensitizer-free alternative suitable for infection control in real-world clinical and laboratory settings.

In addition to its safety advantages, the visible light approach offers clear practical benefits for implementation in operational environments.

In real-world environments, factors such as surface reflectance, geometric shadowing, and material properties may influence local irradiance and thus the efficiency of visible light-based photoinactivation. However, previous studies have shown that violet-blue light retains measurable antimicrobial activity even under indirect or diffuse illumination (Maclean et al. 2014; Murdoch et al. 2012; Hessling et al. 2017). Reflective materials can enhance light distribution (Amodeo et al. 2022), and unlike UV-C, visible light can partially penetrate transparent barriers such as glass or plastic (Dai et al. 2012). These characteristics support the potential of broad-spectrum visible light to perform effectively in complex healthcare environments, although dedicated field studies are warranted to quantify in situ performance.

Taken together, these safety and environmental characteristics support the practical feasibility of implementing visible light-based systems in real-world infection control, while the underlying photodynamic mechanism further strengthens their biological effectiveness.

Another crucial aspect demonstrated in this study is that photoinactivation occurred without the use of external photosensitizers. Our findings confirm that Mtb is also highly susceptible to visible light photoinactivation, even without photosensitizer additives and at relatively low energy doses. A similar approach using visible-spectrum LED light has already shown promising results against viral pathogens, including SARS-CoV-2, demonstrating a viral inactivation without the need for photosensitizers (De Santis et al. 2021; Rathnasinghe et al. 2021).

While such compounds can enhance ROS production, their use in real-world applications introduces complications related to toxicity, regulatory compliance, and practical deployment. This effect may be partially explained by the presence of heme-containing enzymes—such as cytochromes, catalases, and peroxidases—within Mtb. These enzymes include heme groups (protoporphyrin IX with Fe^2^⁺) that absorb light in the Soret band (around 400–420 nm), facilitating intracellular ROS generation upon illumination**.** The exclusive reliance on chromophores naturally present within microbial cells simplifies implementation and increases safety, making the tested system ideal for clinical environments where continuous and unobtrusive protection is necessary.

Finally, the spectral emission of the tested LED module enables seamless integration into existing lighting systems. This feature allows both experimental setups and real-world installations to operate under ambient conditions that accurately reflect actual usage scenarios, in contrast to previous studies that utilized artificial, monochromatic systems with limited translational relevance. Consequently, the devices serve the dual purpose of providing antimicrobial activity while functioning as standard lighting fixtures.

Nevertheless, several limitations should be acknowledged. First, this study relied exclusively on agar-based CFU enumeration, which may not detect viable but non-culturable (VBNC) or dormant bacilli. Although Middlebrook 7H11 is a standard and selective medium for Mtb isolation, future studies will include parallel liquid culture assays to enhance the detection of persistent forms. Second, because all experiments were performed under BSL-3 containment, the design was intentionally restricted to agar-based dose–response assays to ensure biosafety and metrological control. This approach does not fully reproduce surface-level exposure conditions; therefore, subsequent validation phases will include representative materials such as glass, stainless steel, and medical-grade polymers to assess photoinactivation under realistic environmental settings. Third, future work will also include benchmarking against conventional UV-C systems under biosafety-compliant conditions to enable direct comparative analyses. Finally, although the mechanistic role of photo-induced ROS is supported by the literature, direct ROS detection was beyond the scope of this study; dedicated assays and scavenger-based controls will be implemented in future investigations to experimentally confirm the oxidative pathways underlying Mtb inactivation.

In addition to the aspects discussed above, further research should aim to expand the experimental framework by testing multiple Mtb strains, including drug-resistant isolates, to confirm the generalizability of the observed effects. Moreover, investigations under different metabolic conditions—such as hypoxia or nutrient deprivation—and on diverse surface materials (e.g., glass, plastic, and fabric) representative of real environments would be valuable. Long-term or repeated exposure experiments under realistic illumination geometries are also recommended to assess cumulative effects and the practical applicability of visible light-based disinfection systems.

## Conclusion

This study confirms that visible light in the 400–420 nm range can effectively reduce Mtb viability, achieving more than a 2-log reduction at a dose of approximately 1.0 J/cm^2^, depending on exposure time and distance. These results highlight a promising, non-chemical, and photosensitizer-free approach to microbial inactivation that complements traditional disinfection methods. Owing to its intrinsic photophysical mechanism, visible light-based photoinactivation can overcome some limitations of chemical agents, particularly in real-world environments where surface irregularities, organic residues, or material incompatibility may compromise conventional disinfection efficacy.

Unlike UV or chemically mediated disinfection strategies, this approach combines effective antimicrobial performance with a high safety profile, allowing for continuous operation in occupied environments without requiring protective equipment. To the best of our knowledge, this work provides the first demonstration of visible-light-mediated inactivation of Mtb strain directly isolated from human patient under biosafety level-3 (BSL-3) conditions, using a clinically deployable light source safe for human exposure. These findings pave the way for scalable, light-driven decontamination strategies applicable to healthcare and community settings and contribute to the broader effort to counter antimicrobial resistance through non-antibiotic technologies.

Taken together, these findings provide a robust scientific foundation for the development and translational implementation of visible-light-based antimicrobial technologies in healthcare environments, particularly where conventional approaches are impractical or insufficient.

## Data Availability

All data supporting the findings of this study are available within the article.
